# Foliimonas ilicis gen. nov., sp. nov., a carbon monoxide-oxidizing bacterium belonging to a novel genus of the family Phyllobacteriaceae isolated from leaves of Ilex aquifolium

**DOI:** 10.1099/ijsem.0.006953

**Published:** 2025-11-03

**Authors:** Sinchan Banerjee, András Táncsics, Zeqin Wu, Tudor Stafioiu, Jiacheng Gao, Erika Tóth, Erzsébet Baka, Gary Bending, Hendrik Schäfer

**Affiliations:** 1 School of Life Sciences, University of Warwick, Coventry, UK; 2 Department of Molecular Ecology, Hungarian University of Agriculture and Life Sciences, Gödöllő, Hungary; 3 Department of Microbiology, Eötvös Loránd University, Budapest, Hungary

**Keywords:** carbon monoxide, genus, *Ilex aquifolium*, leaf, oxidation, phyllosphere

## Abstract

A novel carbon monoxide (CO)-oxidizing bacterial strain designated as SB112^T^ was enriched and isolated from *Ilex aquifolium* leaves from Tocil Wood Nature Reserve in Coventry, UK. The strain was Gram reaction-negative, aerobic, rod-shaped, motile with a polar flagellum and non-spore-forming. Growth of strain SB112^T^ was observed at 10–45 °C, pH 6.0–12.0 and NaCl concentrations of 1–3%. The genomic DNA G+C content was 58.3 mol%, and the major fatty acids (>10%) of strain SB112^T^ were C_18 : 1_ ω7c, C_18 : 1_ ω7c 11-methyl and C_19 : 0_ cyclo ω7c. Major polar lipids were phosphatidylcholine, diphosphatidylglycerol, phosphatidylglycerol and a phospholipid. Strain SB112ᵀ contains ubiquinone-10 as the major respiratory quinone. Phylogenetic analysis based on 16S rRNA gene sequences showed that strain SB112^T^ formed a separate lineage within the family *Phyllobacteriaceae*, showing sequence identities of 97.7%, 97.6% and 97.5%, with its closest relatives *Aminobacter niigataensis*, *Aminobacter aminovorans* and *Mesorhizobium plurifarium*, respectively. Phylogenomic analyses using whole-genome sequences consistently placed this strain within the family *Phyllobacteriaceae*. However, its phylogenetic position did not correspond to any known genus within this family. The genome of strain SB112^T^ was found to possess the form II *coxL* gene, which encodes the large subunit of the CO dehydrogenase and potentially enables CO oxidation. The average nucleotide identity and digital DNA–DNA hybridization with members of closely related genera yielded values below the thresholds for prokaryotic species delineation (95–96 and 70%, respectively). Based on the phenotypic, chemotaxonomic, phylogenetic, genomic and physiological properties, strain SB112^T^ is considered to represent a novel species of a new genus *Foliimonas* within the family *Phyllobacteriaceae* for which the name *Foliimonas ilicis* gen. nov., sp. nov. is proposed. The type of strain is SB112^T^ (=LMG 33802^T^, =NCAIM B.02691^T^).

## Introduction

The family *Phyllobacteriaceae*, a member of the order *Rhizobiales*, belongs to the class *Alphaproteobacteria* and comprises 19 validly published genera as of January 2025, including *Mesorhizobium* and *Aminobacter*, according to the List of Prokaryotic names with standing in Nomenclature (https://lpsn.dsmz.de/family/phyllobacteriaceae). The family *Phyllobacteriaceae* was first proposed by Mergaert and Swings in 2005 [1]. Members of this family are widely recognized for their associations with plants and their roles in nitrogen fixation, nutrient cycling and bioremediation. They have been isolated from diverse environments such as soil, water and plants [2,3]. Strains belonging to the family *Phyllobacteriaceae* are usually Gram-stain-negative, rod-shaped, aerobic, non-spore forming and non-motile or motile utilizing polar, subpolar or lateral flagella. Ubiquinone-10 (Q-10) is the predominant isoprenoid quinone, and *Phyllobacteriaceae* have high (>50.0 mol%) DNA G+C content [4]. Among the several genera belonging to the family *Phyllobacteriaceae*, certain species of *Mesorhizobium* and *Phyllobacterium* are known to exhibit plant growth-promoting properties [5]. The genus *Mesorhizobium* comprises several species that are primarily associated with the rhizosphere of leguminous plants, where they may form root nodules and engage in symbiotic nitrogen fixation. Some members have been reported to be capable of the degradation of hazardous pollutants [6,7]. Species such as *Mesorhizobium amorphae* and *Mesorhizobium loti* are well-studied for their role in fixing nitrogen in exchange for carbon compounds from the host plant [3]. *Mesorhizobium* is phylogenetically closely related to other nitrogen-fixing genera like *Rhizobium*, and it plays a critical role in enhancing soil fertility and supporting leguminous crop production [8]. In contrast, *Aminobacter* species, such as *Aminobacter aminovorans*, are typically free-living in soil and groundwater, where they can oxidize a wide range of organic compounds, which distinguishes them from *Mesorhizobium* [9], although some *Aminobacter* spp. also possess nitrogen-fixing capabilities and nodulate legumes [10]. Moreover, *Aminobacter carboxidus* was reported to grow chemolithotrophically using carbon monoxide (CO), highlighting the role of the *Aminobacter* genus in nutrient cycling and bioremediation [2,11, 12]. The phyllosphere, the aerial part of plants, represents a distinct microbial habitat exposed to fluctuating environmental conditions, including ultraviolet radiation, nutrient limitation and pollutants. The phyllosphere microbiome can significantly influence plant health and global biogeochemical cycles [13]. During the study of CO-oxidizing phyllosphere microbiota of holly (*Ilex aquifolium*) tree leaves, a novel bacterial strain, SB112^T^, was isolated. This strain was capable of CO oxidation, a metabolic trait contributing to atmospheric carbon turnover and microbial survival under nutrient-limited conditions [11]. CO is one of the most common air pollutants and is highly toxic to humans and animals. Most of the CO generated by natural and man-made sources is removed by biological and non-biological mechanisms, resulting in atmospheric mixing ratios ranging from 40 ppbv to 500 ppbv, or where impacted by biomass burning and anthropogenic pollution, up to several ppm [14]. It has been reported that diverse CO-oxidizing micro-organisms are both abundant and active in plant-associated microbial communities, contributing to the removal of CO [15]. The phyllosphere of holly was previously identified as a habitat for CO-degrading bacteria [16]. In the current work, strain SB112^T^ was isolated from a CO-degrading enrichment culture that had been inoculated with holly leaf wash. Phylogenetic analysis based on 16S rRNA gene sequences placed strain SB112^T^ within the family *Phyllobacteriaceae*, with its closest relatives being members of the genera *Mesorhizobium* and *Aminobacter*. However, comprehensive polyphasic analysis, including genomic, chemotaxonomic and phenotypic characterization, revealed that strain SB112^T^ represents a distinct lineage.

## Methods

### Isolation of strain

Leaf samples of holly (*I. aquifolium*), a common evergreen tree species in the British Isles and Western Europe, were collected from the Tocil Wood Nature Reserve, Coventry, UK (52° 22′ 36′ N 1° 33′ 23′ W). Phyllosphere microbial communities were collected by adapting a leaf-washing method by Atamna-Ismaeel *et al.* [17]. Leaf wash filters were used to set up enrichments to determine the CO oxidation potential of the enriched phyllosphere microbial community. Enrichments were set up in 125 ml crimp-sealed serum vials with 25 ml of sterile mineral salts medium [18], with 0.25 g l^−1^ of yeast extract (to promote the heterotrophic oxidation of CO). CO (≥99%; Sigma-Aldrich, USA) at a concentration of approximately 100,000 ppmv (10% v/v) was injected into the headspace. The enrichment vials were incubated at 25 °C and 100 r.p.m. in a shaking incubator. The CO levels in the headspace were measured every 2 days using an Agilent 6890 N gas chromatograph equipped with an FID and methanizer (GC; Agilent Technologies, California, USA). When the CO concentration dropped below the detection limit, 1 ml of the culture from the enrichment vial was serially diluted with 0.85% (w/v) sterile saline solution. The diluted suspension was spread on LB agar (DSM medium No. 381) and incubated at 25 °C for 5 days for the isolation of bacterial strains. After incubation, multiple colonies were isolated, strains were then purified by repeated streaking on the same medium and, afterwards, their purity and identity were checked by sequencing their 16S rRNA gene. Genomic DNA of strain SB112^T^ was isolated using the DNeasy^®^ UltraClean^®^ Microbial Kit (Qiagen, Hilden, Germany) as per the manufacturer’s instructions. For taxonomic identification of isolates, the 16S rRNA gene was amplified using the primers 27f (5′-AGAGTTTGATCCTGGCTCAG-3′) and 1492r (5′-TACGGYTACCTTGTTACGACTT-3′) [19]. Strain SB112ᵀ was routinely maintained aerobically on LB agar at 25 °C, while for long-term preservation, it was stored at –80 °C in LB supplemented with 30% (v/v) glycerol. This study describes strain SB112^T^ as a novel species and genus, *Foliimonas ilicis* gen. nov., sp. nov. Detailed genomic and metabolic analyses were performed to elucidate its taxonomic and functional characteristics. The combination of its unique phylogenetic position, metabolic versatility and ecological significance in the phyllosphere highlights its novelty.

### 16S rRNA-based phylogeny

For the comprehensive identification of SB112ᵀ, a homology search using the almost complete (1,491 bp) 16S rRNA gene sequence of SB112ᵀ as a query sequence was performed using the EzBioCloud database (https://www.ezbiocloud.net/identify) [20] to determine an approximate phylogenetic affiliation. The 16S rRNA gene homology search resulted in the identification of 40 known species with significant similarity with SB112ᵀ. Afterwards, the 16S rRNA gene sequences of the species that showed high DNA–DNA hybridization (dDDH)/average nucleotide identity (ANI) values with SB112ᵀ in genome analyses performed using the Type Strain Genome Server (TYGS) were acquired from GenBank. Multiple sequence alignment was performed using clustal W with default parameters, and evolutionary distances were calculated based on Kimura’s two-parameter model. Neighbour-joining (NJ), maximum-likelihood (ML) and maximum-parsimony (MP) phylogenetic trees were constructed using mega 11 [21,24]. Tree topologies and distances were evaluated by standard bootstrap analysis based on 1,000 replicates.

### Whole genome-based phylogenomic and functional analysis

For whole-genome sequencing of the isolate SB112^T^, the strain was cultured on an LB plate for 2 days at 25 °C before harvesting biomass, which was then sent to MicrobesNG (Birmingham, UK) where DNA was extracted and sequenced using the Illumina HiSeq platform (250 bp paired-end protocol). Adapters were trimmed, and reads were assembled by MicrobesNG using Trimmomatic [25] and SPAdes version 3.7 [26], respectively. The draft whole-genome sequence was analysed for contamination and completeness by CheckM and ContEst16S tools [27,28]. Genome comparison of SB112ᵀ with eight other strains, including the closest type strains – ‘*Mesorhizobium zhangyense*’ CGMCC 1.15528^T^ (JAAKZG000000000), *A. aminovorans* DSM 7048^T^ (SLZO00000000), *M. loti* DSM 2626^T^ (QGGH00000000), *Mesorhizobium plurifarium* ORS 1032^T^ (CCND00000000), *Mesorhizobium robiniae* DSM 100022^T^ (JBEPMC000000000), *Mesorhizobium shonense* DSM 29846^T^ (JBEPLM000000000), *Mesorhizobium silamurunense* CCBAU 01550^T^ (JHQR00000000) and *Mesorhizobium waimense* ICMP 19557^T^ (QZWZ01000000) – was carried out using formula 2 of the Genome-to-Genome Distance Calculator 3.0 (https://ggdc.dsmz.de/ggdc.php#) and the OAT software [29,31]. The reference genomes for comparison purposes were retrieved from the GenBank database (http://www.ncbi.nlm.nih.gov/genbank/) to determine OrthoANI and dDDH values. Furthermore, to gain preliminary insights into the taxonomic affiliation of SB112ᵀ through genome analyses, a phylogenomic tree was constructed on the TYGS platform [32] with the Genome blast Distance Phylogeny method using the FastME 2.1.6.1 tool [33]. Additionally, another phylogenomic tree was constructed using a concatenated alignment of 92 core genes with UBCG software [34] by applying the FastTree algorithm [35]. The assembled genome data were annotated using both the Prokaryotic Genome Annotation Pipeline [36] and the Rapid Annotation Subsystem Technology (RAST) server [37]. Orthologous gene cluster comparisons between strain SB112^T^ and the two closely related reference type strains (*A. aminovorans* and *M. loti*) were conducted using OrthoVenn3 [38]. Pairwise core-proteome average amino acid identity (cpAAI) analysis was performed for the *F. ilicis* SB112^T^ using the database and pipeline as described by Kuzmanović *et al*. [39] supplemented with the closest relatives of *F. ilicis* SB112^T^, namely, *M. zhangyense* CGMCC 1.15528^T^, *A. aminovorans* DSM 7048^T^, *M. loti* DSM 7048^T^, *M. plurifarium* ORS1032^T^, *M. robiniae* DSM 100022^T^, *M. shonense* DSM 29846^T^, *M. silamurunense* CCBAU 01550^T^ and *M. waimense* ICMP 19557^T^. The cpAAI analyses were conducted using the publicly available pipeline specifically developed for *Rhizobiaceae* (https://github.com/flass/cpAAI_Rhizobiaceae). Visualization and comparative analyses were performed in RStudio (version 2024.04.2; RStudio Team, Boston, MA, USA) employing the phylo.heatmap function (R Core Team) to generate heatmap representations, while Microsoft Excel was used for supplementary data handling. Whole-genome relatedness of *F. ilicis* SB112^T^ was assessed using FastAAI [40], which estimates average amino acid identity (AAI) by comparing conserved universal protein tetramers across genomes. The genome of *F. ilicis* SB112^T^ was compared against selected reference genomes representing close phylogenetic relatedness. These reference genomes were the same as those used in the OrthoANI analysis to ensure consistency across whole-genome relatedness comparisons. For each pairwise comparison, the average Jaccard similarity of shared conserved protein sequences (SCPs) was calculated across 78 universal SCPs, and an AAI estimate was derived automatically by FastAAI. The virulence factors and pathogenicity potential of the strains were determined by VirulenceFinder 2.0 [41] and PathogenFinder 1.1 [42]. Genes involved in secondary metabolism were predicted using the antibiotics and Secondary Metabolite Analysis Shell (antiSMASH) version 5.0 [43]. Comprehensive Antibiotic Resistance Database (CARD) [44] was used to predict antimicrobial resistance genes.

### Morphological, physiological, biochemical and chemotaxonomic analysis

For morphological, physiological, ecological and chemotaxonomic study, reference strains of *A. aminovorans* DSM 7048ᵀ and *M. loti* LMG 6125ᵀ were obtained from BCCM/LMG Bacteria Collection (Belgium) and were studied under the same laboratory conditions along with strain SB112ᵀ. Cell size, shape and arrangement of strain SB112ᵀ were studied by native preparations and by Gram-staining according to Claus [45]. The cell morphology and flagellation type of strain SB112ᵀ were investigated during the exponential growth phase using transmission electron microscopy (JEOL2100 Plus). Cells for electron microscopy were negatively stained according to the procedure of [46]. Briefly, 5 µl of purified bacterial culture was added to a glow-discharged carbon/formvar-coated 300-mesh copper TEM grid (EM Resolutions, Sheffield, UK) and stained with 2% uranyl acetate for 1 min. The grid was imaged on the JEOL2100Plus electron microscope with a Gatan OneView camera. The following physiological and biochemical tests were performed according to the protocols of Barrow and Feltham [47]: urease activity; Baird–Parker’s phosphatase activity; production of hydrogen sulphide from cysteine and indole from tryptophan; and hydrolysis of casein, gelatine, aesculin and Tween 80. Catalase activity was determined by bubble production with H_2_O_2_ (3%, v/v), and oxidase activity was tested using 1% (w/v) tetramethyl-p-phenylenediamine oxalate. Growth at different temperatures (from 4 to 45 °C) was tested using LB broth medium. The optimum pH for growth was estimated using LB broth, and the pH of the medium was adjusted to 4.0–12.0 (at intervals of 1 pH unit) prior to autoclaving using citrate/NaH_2_PO_4_ buffer (0.1 M, for pH range 4.0–5.0), phosphate buffer (0.1 M, for pH range 6.0–7.0), Tris buffer (0.2 M, for pH range 8.0–10.0) and NaOH (5 M, for pH range 10.0–12.0). Salt tolerance was assessed by inoculating the strain into LB broth supplemented with 0–5% (w/v) NaCl at 1% intervals. During pH and NaCl tolerance tests, growth was determined by measuring OD at 600 nm. API 50CH, API 20NE and API ZYM strips (bioMérieux) were used to evaluate physiological and biochemical characteristics according to the manufacturer’s instructions. Growth under anaerobic conditions was determined in LB broth medium with and without the addition of 0.15% (w/v) KNO_3_ at 28 °C. To ensure anaerobic conditions, 100 ml serum bottles (Glasgerätebau Ochs) with 50 ml LB broth were crimp-sealed and purged with nitrogen under sterile conditions as described earlier [18]. The whole-cell fatty acids, respiratory quinones and polar lipids were analysed by DSMZ Services, Leibniz-Institut DSMZ - Deutsche Sammlung von Mikroorganismen und Zellkulturen GmbH (Braunschweig, Germany). Briefly, cellular fatty acids were converted into fatty acid methyl esters (FAMEs) by saponification, methylation and extraction by applying the protocol of Sasser [48]. Subsequently, the FAME mixtures were separated by GC and detected by a flame ionization detector. In subsequent analysis, fatty acids were identified by a GC-MS analysis, on an Agilent GC-MS 7000D system [49]. The polar lipids and respiratory quinones were extracted based on the protocol modified after Bligh and Dyer [50]. The separation and detection of the total lipid material and functional groups were performed as described by Banerjee *et al*. [51]. The respiratory quinones were analysed by LDC Analytical (Thermo Separation Products) HPLC apparatus fitted with a reverse-phase column (Macherey-Nagel; 2.125 mm, 3 µm, RP18) using methanol: heptane 9 : 1 (v/v) as eluant. The ability of strain SB112^T^ to oxidize CO was evaluated using GC. Cultures were grown in serum vials containing minimal media with 100 ppmv CO and yeast extract (250 mg l^−1^), and CO concentrations were monitored using an Agilent 6890 N gas chromatograph equipped with a flame ionization detector and a methanizer as described previously [16].

## Results and discussion

**Fig. 1.**
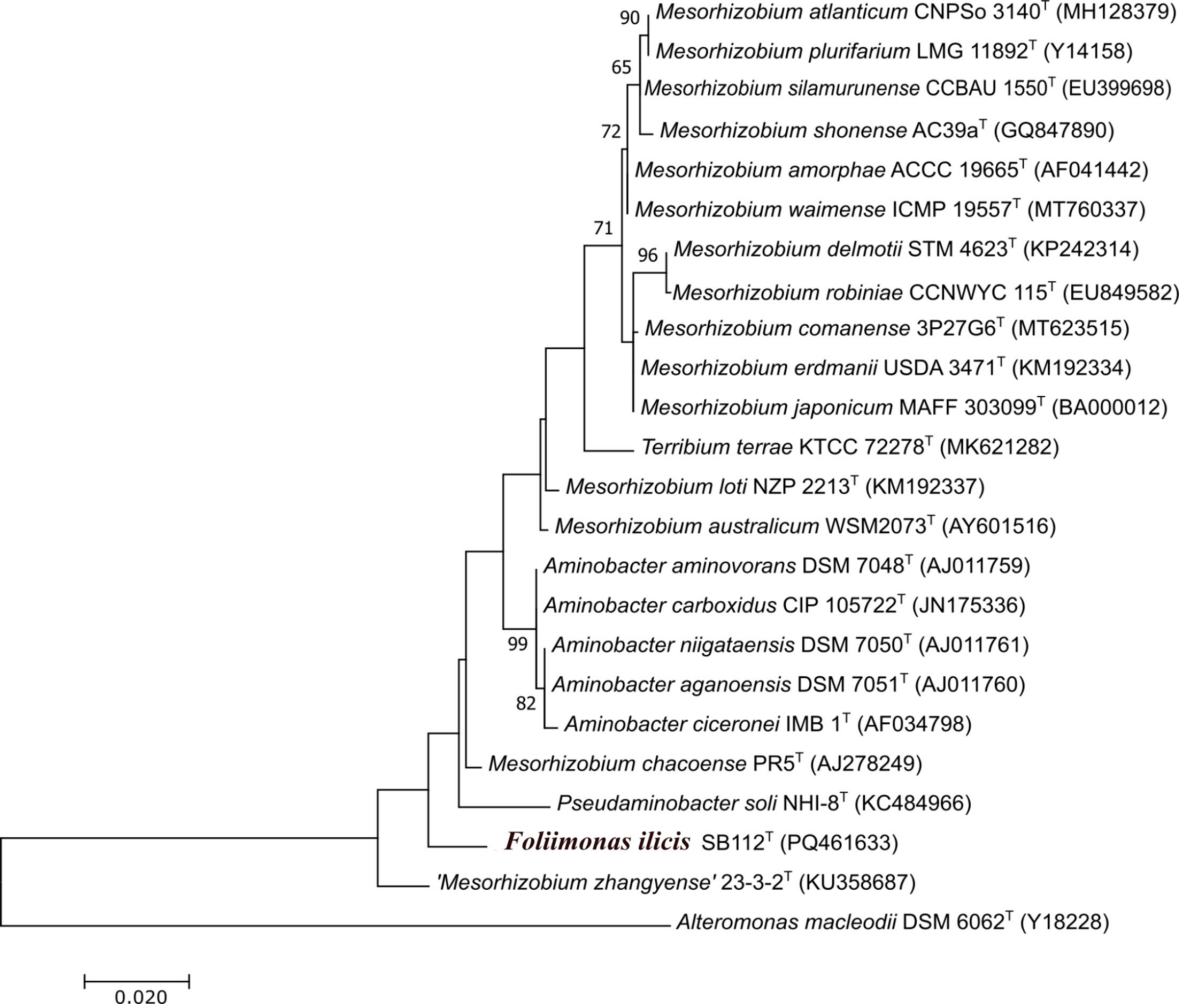
ML tree based on 16S rRNA gene sequences showing the phylogenetic relationships between *F. ilicis* SB 112^T^ and related taxa. Bootstrap values are shown as percentages of 1,000 replicates. *Alteromonas macleodii* DSM 6062^T^ was used to root the tree. Bar, 0.02 substitution per nucleotide position.

**Fig. 2.**
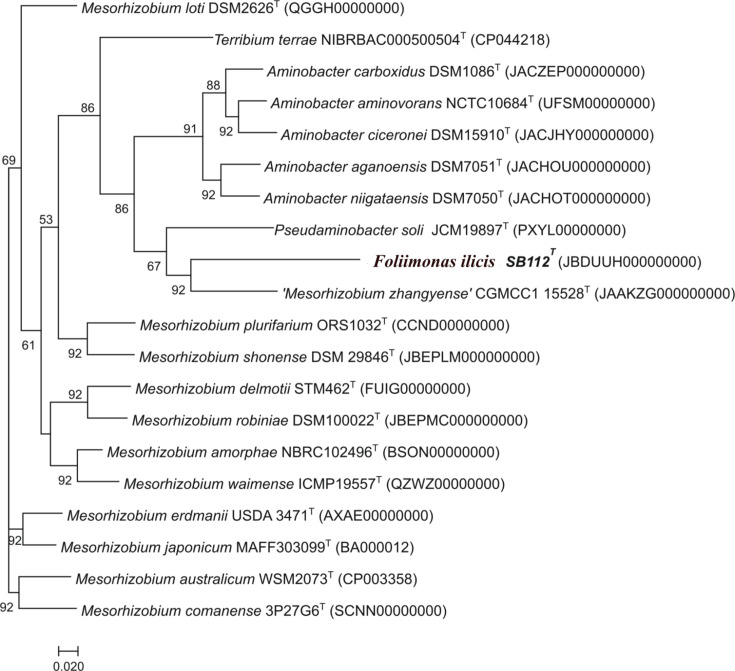
Phylogenomic tree constructed using UBCGs (concatenated alignment of 92 core genes) showing the phylogenetic position of *F. ilicis* SB 112^T^. For inferring the tree, the FastTree algorithm was used. Bar, 0.02 substitution per nucleotide position.

**Fig. 3.**
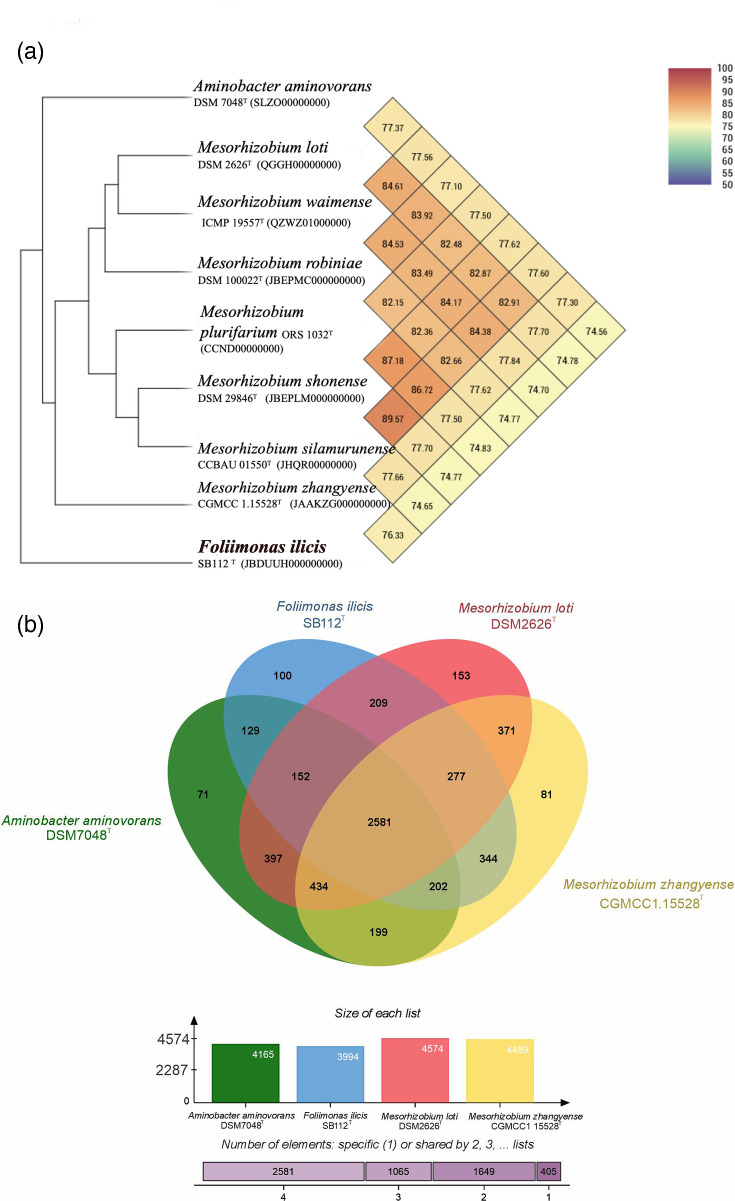
(a) Heatmap generated with OrthoANI values between *F. ilicis* SB112^T^ and other closely related type strains of the genera *Mesorhizobium* and *Aminobacter*. Venn diagram of orthologous genes of *F. ilicis* SB112^T^ and the closest reference strains.

Phylogenetic analysis of the almost complete 16S rRNA gene (1,491 bp) sequence of SB112^T^ revealed that the strain belonged to the phylum ‘Pseudomonadota’, in which it formed a distinct phyletic lineage within the family *Phyllobacteriaceae* showing similar distances to different genera/species within the family but not clearly classifying into any of them based on 16S rRNA sequence similarities (Fig. 1). The 16S rRNA gene sequence similarity indicated that strain SB112ᵀ was most closely related to *Aminobacter niigataensis* DSM 7050^T^ and *Aminobacter aganoensis* DSM 7051^T^ (both 97.7% similarity) followed by *A. aminovorans* DSM 7048^T^, *Mesorhizobium chacoense* PR5^T^, *Aminobacter ciceronei* IMB-1^T^, *M. shonense* AC39a^T^ (all 97.6%), *M. plurifarium* LMG 11892^T^ and *Mesorhizobium atlanticum* CNPSo 3140^T^ (both 97.5%); however, phylogenetic analysis based on 16S rRNA gene sequences suggested that strain SB112ᵀ did not belong to any existing genera but formed a distinct phylogenetic lineage within the family *Phyllobacteriaceae* in the ML-based analysis (Fig. 1). Phylogenetic trees reconstructed by the NJ tree (Fig. S1, available in the online Supplementary Material) or MP (Fig. S2) supported this observation. Phylogenomic analysis using UBCG on a concatenated alignment of 92 core genes (Fig. 2) also demonstrated that strain SB112^T^ represents a separate lineage, clearly indicating that strain SB112ᵀ is a novel member within the family *Phyllobacteriaceae* at the genus and species level. Hence, the two type species of the closest genera, *A. aminovorans* DSM 7048ᵀ and *M. loti* DSM 2626^T^, were included in further analyses as reference strains. Analyses with CheckM and ContEst16S revealed that the SB112^T^ genome sequence has 99.02% completeness and 1.07% contamination. The draft genome of the strain has been assembled into 60 contigs with a total length of 5,747,759 bp, comprising 5,958 genes of which 5,903 are protein CDSs, 49 encode tRNA genes and 6 encode rRNA genes. The SB112^T^ genome has an average G+C content of 58.3 mol%. For species delineation, the accepted threshold is at least 70% DNA–DNA relatedness and 95–96% OrthoANI similarity [31,52]. The ANI and dDDH analysis among SB112^T^ with its closest relatives resulted in dDDH values ranging between 19.9% and 21.2% and OrthoANI values ranging from 74.55% to 76.32% (Fig. 3a). The highest dDDH value among the closest relatives was observed with ‘*Mesorhizobium zhangyense*’ CGMCC 1.15528^T^, which was 21.2% (Table S1). However, this value was markedly lower than the prokaryotic species delineation cutoff [53]. Similarly, OrthoANI and ANI algorithm using blast values also showed ‘*Mesorhizobium zhangyense*’ CGMCC 1.15528^T^ as the closest match (Table S1), though the values (76.32% and 74.91%, respectively) remained significantly below the threshold (95–96% ANI) for a strain to be classified as the same species [53,54]. The ANI and dDDH values obtained for the type strains of the respective species are presented in Table S1. To further evaluate the taxonomic position of *F*. *ilicis* SB112^T^, we performed a cpAAI analysis following the framework of Kuzmanović *et al.* [39]. Their study provided a reference dataset of representative members across the family *Rhizobiaceae*, including type strains of *Rhizobium*, *Agrobacterium*, *Pseudorhizobium*, *Neorhizobium*, *Allorhizobium*, *Ciceribacter*, *Ensifer*/*Sinorhizobium*, *Pararhizobium*, *Shinella*, *Hoeflea*, *Martelella*, *Georhizobium* and others. We supplemented this dataset with the closest relatives of *F. ilicis* SB112^T^, namely, *M. zhangyense*, *A. aminovorans*, *M. loti*, *M. plurifarium*, *M. robiniae*, *M. shonense*, *M. silamurunense* and *M. waimense*. The cpAAI values obtained for *F. ilicis* SB112^T^ ranged from 65.6% to 83.7% (Table S2). The highest similarity (83.7%) was observed with *M. zhangyense* CGMCC 1.15528^T^, while all other relatives showed values below this (Fig. S3). Furthermore, the FastAAI analysis showed that *F. ilicis* SB112^T^ is most closely related to *M. zhangyense* with an estimated AAI of 67.13%. Other *Mesorhizobium* species, including *M. loti*, *M. robiniae*, *M. plurifarium*, *M. shonense*, *M. silamurunense* and *M. waimense*, exhibited similar AAI estimates ranging from ~64.3% to 64.9%. (Table S3) The closest non-*Mesorhizobium* comparison, *A. aminovorans*, showed an AAI of 65.25%. Genomic comparisons clearly distinguished strain SB112ᵀ from the described genera of the *Rhizobiaceae*. ANI values with the closest relatives ranged from 74.5% to 76.3%, below the genus thresholds proposed by Kuzmanović *et al*. (78.5%). Similarly, FastAAI values (64.3–67.1%) fell outside the proposed genus-level range (suggested threshold 76.5%), while dDDH values (19.9–21.2%) were far below the 70% species cutoff. Core-proteome AAI analysis yielded values of 65.6–83.8% with members of the family, with the closest relative (‘*Mesorhizobium zhangyense*’ CGMCC 1.15528ᵀ – 83.8%) still below the genus boundary of 86%. Collectively, these data provide robust evidence that SB112ᵀ represents a novel genus within the *Rhizobiaceae*. The genome sequencing data obtained for SB112ᵀ were deposited in DDBJ/ENA/GenBank under the accession number JBDUUH000000000. The RAST annotation of the SB112^T^ genome sequence showed 336 subsystem features that provide a functional overview of the genome. These subsystems contain genes or gene clusters that are involved in different cellular processes such as metabolism, transport and regulation (Fig S4) [1]. Additionally, the KEGG annotation predicted that the SB112^T^ genome encodes 134 pathway modules categorized into the following functional subcategories: amino acid metabolism, biosynthesis of polyketides and non-ribosomal peptides, biosynthesis of secondary metabolites, carbohydrate metabolism, energy metabolism, glycan biosynthesis and metabolism, immune system, lipid metabolism, metabolism of cofactors and vitamins, metabolism of other amino acids, nucleotide metabolism, signal transduction, translation and xenobiotics biodegradation and metabolism. PathogenFinder and VirulenceFinder predicted that strain SB112^T^ was a non-pathogenic strain for humans. The CARD tool did not detect any perfect hits for antibiotic resistance genes. The annotation results revealed that the SB112^T^ genome harbours several genes encoding functional proteins with potential ecological and biotechnological significance. The genome comparative data generated in the Venn diagram (Fig. 3b) revealed that 2,555 orthologous genes were common across all analysed genomes, that is, strain SB112^T^ and the three closest type strains (*A. aminovorans* DSM 7048ᵀ, *M. loti* DSM 2626ᵀ and CGMCC 1.15528ᵀ), and 82 genes were unique to strain SB112^T^ (Fig. 3b). According to the RAST annotations, the presence of cytochrome *c* oxidase polypeptides I, II and III (complex IV) and NADH-ubiquinone oxidoreductase (complex I) subunit A, which are crucial elements of aerobic energy metabolism, validates that SB112^T^ is an aerobe [55]. Additionally, the genome of SB112^T^ contains genes encoding enzymes responsible for nitrite ammonification, specifically the large and small subunits of nitrite reductase. However, no genes assigned to the CO₂ fixation subsystem were found, and the gene encoding ribulose-1,5-bisphosphate carboxylase/oxygenase (Rubisco) was absent, indicating the lack of this CO₂ fixation pathway [56]. Furthermore, the genome also contains genes that encode enzymes for the complete tricarboxylic acid cycle [57]. The genome assembly of strain SB112^T^ contained genes encoding form II CO dehydrogenase (CODH; BMS type), which was confirmed by the presence of the active site motif AYRGAGR [11]. The putative form II *coxL* gene of SB112^T^ had the highest similarity (91.79%) to ‘*Mesorhizobium zhangyense*’ CGMCC 1.15528ᵀ xanthine dehydrogenase family protein molybdopterin-binding subunit (WP_165113128.1). The putative CODH gene cluster in SB112^T^ comprises three genes transcribed in the order *coxS* (WP_347971887.1), *coxL* (WP_347971886.1) and *coxM* (WP_347971885.1), the arrangement associated with form II CODH genes, which is in contrast to the typical organization of form I operons in which genes are in the order *coxMSL* [58]. In the absence of a form I CODH, it is likely that the form II CODH is responsible for the oxidation of CO in SB112^T^. In addition, genes encoding glutathione synthase, catalase/peroxidase, hydroperoxidase and cold shock proteins were also detected in the genome sequence of SB112^T^. These proteins are primarily linked to oxidative stress responses and low-temperature adaptation, suggesting that strain SB112^T^ is capable of surviving in an environment with oxidative stress or temperature fluctuation [59,60]. Moreover, the SB112^T^ harbours genes encoding pyrroline-5-carboxylate reductase and synthase associated with proline and glycogen metabolism. This indicates that the strain may contribute to mitigating abiotic stresses in plant-associated environments [61]. On the other hand, genes involved in glycogen metabolism indicate a method for storing and using energy, which may help the strain survive in environments with fluctuating nutritional levels [62]. The presence of genes related to plant growth promotion implies a potential role in plant-microbial interactions. Genes encoding the alpha and beta subunits of tryptophan synthase (WP_347970016.1 and WP_347970015.1) are known to play a role in the synthesis of auxin, a phytohormone that promotes root elongation and overall plant development [63]. The antiSMASH biosynthetic gene cluster analysis of the SB112^T^ genome revealed the presence of biosynthetic gene clusters for *N*-acetylglutaminylglutamine amide (NAGGN), terpenes, NRPS-independent siderophores (NI-siderophores) and homoserine lactones (HSLs). NAGGN is known to play a crucial role in osmotic stress response in bacteria. Its biosynthesis is regulated by a specialized enzymatic pathway, with key genes upregulated under osmotic stress. By enhancing osmoprotection, NAGGN might support the survival and ecological adaptation of phyllosphere bacteria, enabling them to withstand and thrive in fluctuating humidity [64,65]. Terpenes have antimicrobial and signalling properties and can influence microbial communities and trigger plant defence responses, which can enable bacteria to form advantageous relationships with plants [66]. NI-siderophores, such as *IucA*/*IucC*-like compounds, facilitate iron acquisition and may support bacterial colonization in the iron-limited environment while promoting plant growth by improving iron availability [67]. HSLs function as quorum-sensing molecules, regulating biofilm formation and secondary metabolite production, which could play an important role in plant-microbe interaction [68]. SB112ᵀ was able to oxidize CO (data not shown). The initial degradation of 100 ppm CO by strain SB112^T^ was slow (~37 days), but degradation rates increased significantly in subsequent reinjections, likely due to microbial growth and enhanced metabolic activity. These findings suggest that SB112^T^ can oxidize CO at low concentrations; however, further investigation is required to establish whether it is able to oxidize CO at atmospheric concentrations. Overall, the functional gene diversity in strain SB112^T^ hints towards its multifunctional ecological role. These characteristics enhance its adaptability in plant-associated environments while showcasing its potential for biotechnological applications.

**Fig. 4.**
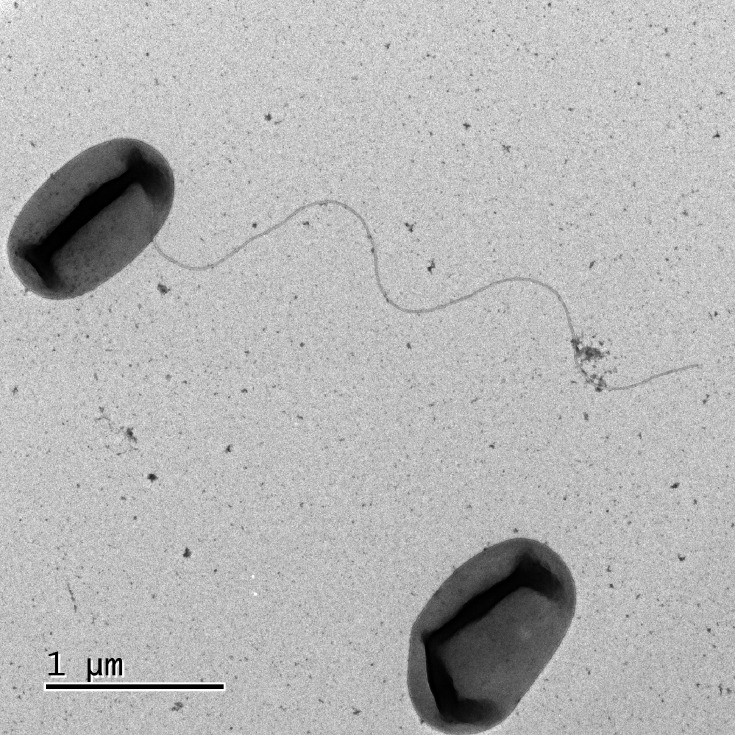
Transmission electron microscopic photograph showing cell morphology and presence of flagella in strain SB112^T^. Bar=1 µm.

SB112^T^ is an aerobic, Gram-stain-negative, rod-shaped bacterium with a polar flagellum. The cell size (mean±sd) is 1.18±0.04 µm×0.68±0.029 µm (Fig. 4). Strain SB112^T^ formed light yellow/cream, slightly convex, smooth, opaque and circular colonies on LB agar plates at 25 °C after 48 h of incubation. SB112^T^ grows well between 20°C and 37°C but not at 4 °C. SB112^T^ can grow across a wide pH range from 6.0 to 12.0 and tolerates up to 3% NaCl (Table 1). Moreover, strain SB112^T^ grew better in saline (1–3% NaCl) than in the salt-free control. Strain SB112^T^ was unable to hydrolyse Tween 80, starch and casein and was positive for catalase and oxidase. In API ZYM tests, SB112^T^ showed positive enzymatic activity for esterase (C4), esterase lipase (C8), leucine arylamidase, acid phosphatase, naphthol-AS-BI-phosphohydrolase, valine arylamidase, cystine arylamidase and trypsin. For API 20NE, strain SB112^T^ showed positive results for urease activity, but it was negative for aesculin ferric citrate (*β*-glucosidase) hydrolysis and aerobic NO_3_^-^ reduction. SB112^T^ assimilated d-glucose, l-arabinose, d-mannitol, d-mannose, potassium gluconate and malic acid. The results of API50 CH tests showed that strain SB112^T^ could metabolize substrates like l-arabinose, d-xylose and xylitol. In the biochemical tests, the strain showed negative results for indole production and the MR-VP test. In the anaerobic growth monitoring experiment, there was weak growth observed after incubation. The detailed comparative characteristics are given in Table 1, with those of the phylogenetically closest species of the genera *Aminobacter* and *Mesorhizobium*. The major fatty acids (>10%) of strain SB112^T^ were C_18 : 1_ ω7c, C_18 : 1_ ω7c 11-methyl and C_19 : 0_ cyclo ω7c. The fatty acid composition of strain SB112^T^ was more similar to that of *M. loti* LMG 6125^T^ than to *A. aminovorans* LMG 2122^T^ (Table 2). However, it should be noted that the ratios of the major fatty acids can vary within wide limits in both *Mesorhizobium* and *Aminobacter* strains [69,70]. Notable differences were observable only in the case of the minor components. Fatty acids iso-C_15 : 0_, iso-C_17 : 0_ and C_18 : 1_ ω7t were detected only in strain SB112^T^, whereas C_18 : 2_ ω8,13c was detected only in the reference strains. Similar to members of the genera *Mesorhizobium* and *Aminobacter*, the major respiratory quinone of strain SB112^T^ was Q-10. Ubiquinone-8 and ubiquinone-9 were detected as minor components at a percentage of 8.9% and 0.2%, respectively. The major polar lipids were phosphatidylcholine, diphosphatidylglycerol, phosphatidylglycerol and a phospholipid. As minor polar lipid components, several unknown aminolipids, phospholipids and lipids were detected (Fig. S5). In conclusion, the taxonomic information provided here supports the candidature of SB112^T^ as a novel species of a new genus in the family *Phyllobacteriaceae*. We propose the name *F. ilicis* gen. nov., sp. nov. for strain SB112^T^.

**Table 1.** Major phenotypic and biochemical characteristics of strain SB112^T^ and other closely related type strains: 1, strain SB112^T^; 2, *M. loti* LMG 6125^T^; 3, *A. aminovorans* LMG 2122^T^. +, positive; w+, weak positive; −, negative. All data were obtained in this study

Characteristics	1	2	3
Isolation source	Leaves of *I. aquifolium*	*Lotus corniculatus nodule*	Garden soil
Genome size (Mb)	5.7	4.9	5.8
DNA G+C content (mol%)	58.3	62.5	63
Temperature range for growth (°C)	10–45	10–37	45
pH range for growth	6–12	6–12	6–12
NaCl tolerance (%, w/v)	1–3	0–2	0–2
Enzyme activities (API ZYM):			
Valine arylamidase	+	−	+
Cystine arylamidase	+	+	−
Acid phosphatase	+	−	+
*α*-Glucosidase	+	−	+
*N*-Acetyl-*β*-glucosaminidase	w+	−	−
Assimilation of (API 20NE):			
Urea (urease)	+	−	+
Aesculin ferric citrate (*β*-glucosidase) hydrolysis	−	−	+
4-Nitrophenyl-*β*-d-galactopyranoside (*β*-galactosidase)	−	+	−
l-Arabinose	+	+	w+
d-Mannose	−	+	−
Potassium gluconate	+	−	w+
Malic acid	+	−	−
Acid production from (API 50 CHB/E):			
d-Xylose	w+	+	+
d-Mannose	−	+	+
Aesculin ferric citrate	−	−	+
d-Cellobiose	−	+	+
d-Maltose	−	+	+
d-Melibiose	−	+	−
d-Trehalose	−	+	+
Xylitol	+	+	−

**Table 2.** Cellular fatty acid compositions of strain SB112^T^ and related species. Taxa: 1, strain SB112^T^; 2, *M. loti* LMG 6125^T^; 3, *A. aminovorans* LMG 2122^T^; data are expressed as percentages of total fatty acids. Fatty acids which were lower than 1.0% in all strains are not shown. All data are from the present study. nd, not detected; TR, trace amount (<1%)

Fatty acid	1	2	3
iso-C_15 : 0_	1.3%	nd	nd
C_16 : 1_ ω7c	4.3%	1.7%	TR
C_16 : 0_	6.1%	5.8%	3.3%
iso-C_17 : 0_	4.8%	nd	TR
C_17 : 1_ ω8c	2.1%	TR	TR
F*C_17 : 1_ ω6c / C_17 : 0_ cyclo ω7c	2.3%	1.4%	nd
C_17 : 0_	2.1%	TR	TR
C_18 : 1_ ω7c	39.0%	33.9%	60.5%
C_18 : 1_ ω7t	1.9%	nd	nd
C_18 : 2_ ω8,13c	nd	1.5	2.2
C_18 : 0_	1.4%	TR	TR
C_18 : 1_ ω7c 11-methyl	19.8%	20.1%	22.1%
*C_19 : 0_ cyclo ω7c/ω6c	10.7%	29.5%	4.2%
C_22 : 1_ ω12c	nd	nd	2.2%

* Double GC-MS peaks of the corresponding fatty acids. According to the naming of the MIDI TSB6 database, C_17 : 1_ ω6c/C_17 : 0_ cyclo ω7c was identified by MIDI as C_17 : 0_ cyclo, while C_19 : 0_ cyclo ω7c/ω6c was identified by MIDI as C_19 : 0_ cyclo ω8c.

## Description of *Foliimonas* gen. nov.

*Foliimonas* gen. nov. (Fo.li.i.mo’nas. L. neut. n. *folium*, leaf; Gr. fem. n. *monas*, a unit or monad; N.L. fem. n. *Foliimonas*, a monad isolated from a leaf).

Cells are rod-shaped, motile, Gram-stain-negative and aerobic. The principal fatty acids are C_18 : 1_ ω7c, C_18 : 1_ ω7c 11-methyl and C_19 : 0_ cyclo ω7c. The major respiratory quinone is Q-10. The major polar lipids are phosphatidylcholine, diphosphatidylglycerol, phosphatidylglycerol and a phospholipid. The genus belongs to the family *Phyllobacteriaceae* within the phylum *Pseudomonadota*. The type species is *F. ilicis*.

## Description of *Foliimonas ilicis* gen. nov., sp. nov.

*Foliimonas ilicis* sp. nov. [i.li’cis. L. gen. n. *ilicis*, of the holly tree (Ilex), referring to its association with holly or a related habitat].

Members belonging to this species are Gram-reaction-negative, aerobic, motile with a polar flagellum and rod-shaped. The cell size (mean±sd) is 1.18±0.04 µm×0.68±0.029 µm. Optimum growth temperature ranges from 20 °C to 37 °C but shows no growth at 4 °C. On LB agar plates, the colonies are light yellow/cream, slightly convex, smooth, opaque and circular after 48 h of incubation at 25 °C. The strain showed positive reaction for oxidase and catalase activity. The strain was negative for the indole test. In API ZYM tests, SB112^T^ showed positive enzymatic activity for esterase (C4), esterase lipase (C8), leucine arylamidase, acid phosphatase, naphthol-AS-BI-phosphohydrolase, valine arylamidase, cystine arylamidase and trypsin. For API 20NE, strain SB112^T^ showed positive results for urease activity. It showed negative reactions for aesculin ferric citrate (*β*-glucosidase) hydrolysis and aerobic NO_3_^-^ reduction. SB112^T^ exhibited assimilation of d-glucose, l-arabinose, d-mannitol, d-mannose, potassium gluconate and malic acid. The results of API50 CH tests showed that strain SB112^T^ could metabolize substrates like l-arabinose, d-xylose and xylitol. In the biochemical tests, the strain showed negative results for indole production and the MR-VP test. The principal fatty acids are C_18 : 1_ ω7c, C_18 : 1_ ω7c 11-methyl and C_19 : 0_ cyclo ω7c. The major respiratory quinone is Q-10. The major polar lipids are phosphatidylcholine, diphosphatidylglycerol, phosphatidylglycerol and a phospholipid. The genome of SB112^T^ has a total length of 5.74 Mb with a DNA G+C content of 58.3 mol%.

SB112^T^ (=LMG 33802^T^, =NCAIM B.02691^T^) was isolated from the leaf of a holly tree (*I. aquifolium*) sampled from Tocil Wood Nature Reserve, Coventry, UK, in 2024 and can oxidize CO. Genome sequencing data for SB112^T^ have been deposited in DDBJ/ENA/GenBank under the accession number JBDUUH000000000. The 16S rRNA gene sequence of SB112^T^ is available under the accession number PQ461633.

## Supplementary material

10.1099/ijsem.0.006953 Uncited Fig. S1.

10.1099/ijsem.0.006953 Uncited Table S1.

## Abbreviations

AAI: average amino acid identity
ANI: average nucleotide identity
antiSMASH: antibiotics and Secondary Metabolite Analysis Shell
CO: carbon monoxide
CODH: CO dehydrogenase
cpAAI: core-proteome average amino acid identity
dDDH: digital DNA–DNA hybridization
FAMEs: fatty acid methyl esters
HSLs: homoserine lactones
LMG: Belgian Coordinated Collections of Microorganisms (BCCM)
ML: maximum-likelihood
MP: maximum-parsimony
NAGGN: *N*-acetylglutaminylglutamine amide
NCAIM: National Collection of Agricultural and Industrial Microorganisms, Hungary
NI-siderophores: NRPS-independent siderophores
NI-siderophores: NRPS-independent siderophores
NJ: neighbour-joining
Q-10: ubiquinone-10
RAST: Rapid Annotation Subsystem Technology
SCPs: shared conserved protein sequences
TYGS: Type Strain Genome Server
