# Supplementary material for: Foliimonas ilicis gen. nov., sp. nov., a carbon monoxide-oxidizing bacterium belonging to a novel genus of the family Phyllobacteriaceae isolated from leaves of Ilex aquifolium
Source: Int J Syst Evol Microbiol. 2025 Nov 3;75(11):006953. doi: 10.1099/ijsem.0.006953 (PMC12582533; doi:10.1099/ijsem.0.006953)
Supplement: Uncited Fig. S1. [file ijsem-75-06953-s001.pdf]

IJSEM supplementary materials:

Foliimonas ilicis gen. nov., sp. nov, a carbon monoxide oxidizing bacterium belonging to a novel genus of the family *Phyllobacteriaceae* isolated from leaves of *Ilex aquifolium*

Sinchan Banerjee[1], András Táncsics[2], Zeqin Wu[1], Tudor Stafioiu[1], Jiacheng Gao[1], Erika Tóth[3], Erzsébet Baka[2], Gary Bending[1], Hendrik Schäfer[1]

1. School of Life Sciences, University of Warwick, Coventry, United Kingdom.

2. Department of Molecular Ecology, Hungarian University of Agriculture and Life Sciences, Gödöllő, Hungary

3 Department of Microbiology, Eötvös Loránd University, Budapest, Hungary

Corresponding author

Sinchan Banerjee, School of Life Sciences, University of Warwick, Coventry, CV4 7AL, United Kingdom.

E-mail: Sinchan.Banerjee@warwick.ac.uk

Fig: S1

Neighbour-Joining tree based on 16S rRNA gene sequences showing the phylogenetic relationships between strain SB112 ^{T} and related taxa. Bootstrap values are shown as percentages of 1000 replicates. *Alteromonas macleodii* DSM 6062 ^{T} was used to root the tree. Bar, 0.02 substitution per nucleotide position

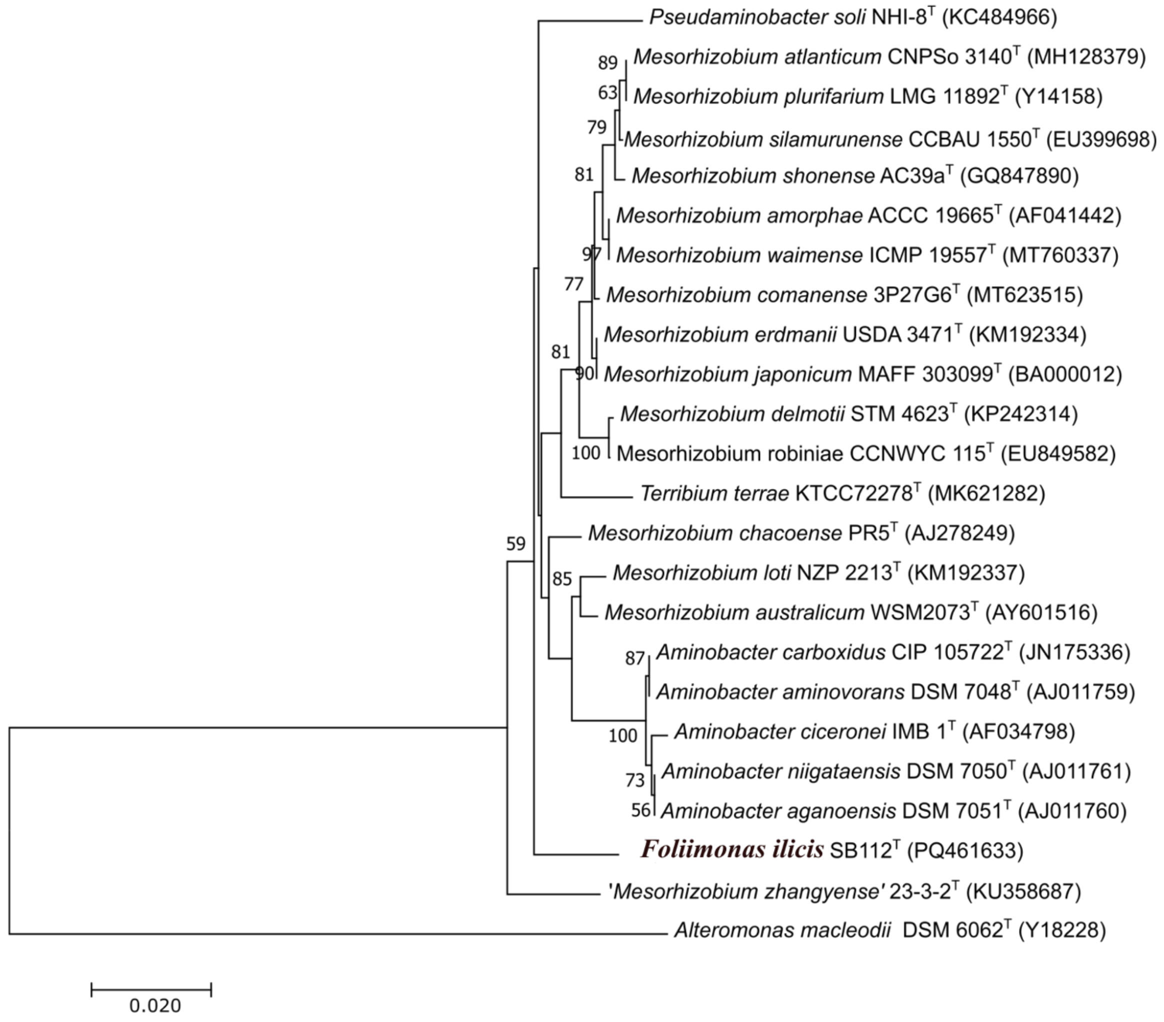

Fig S2:
Maximum parsimony tree based on 16S rRNA gene sequences showing the phylogenetic relationships between strain SB112^{T} and related taxa. Bootstrap values (only >50) are shown as percentages of 1000 replicates. *Alteromonas macleodii* DSM 6062^{T} was used to root the tree.

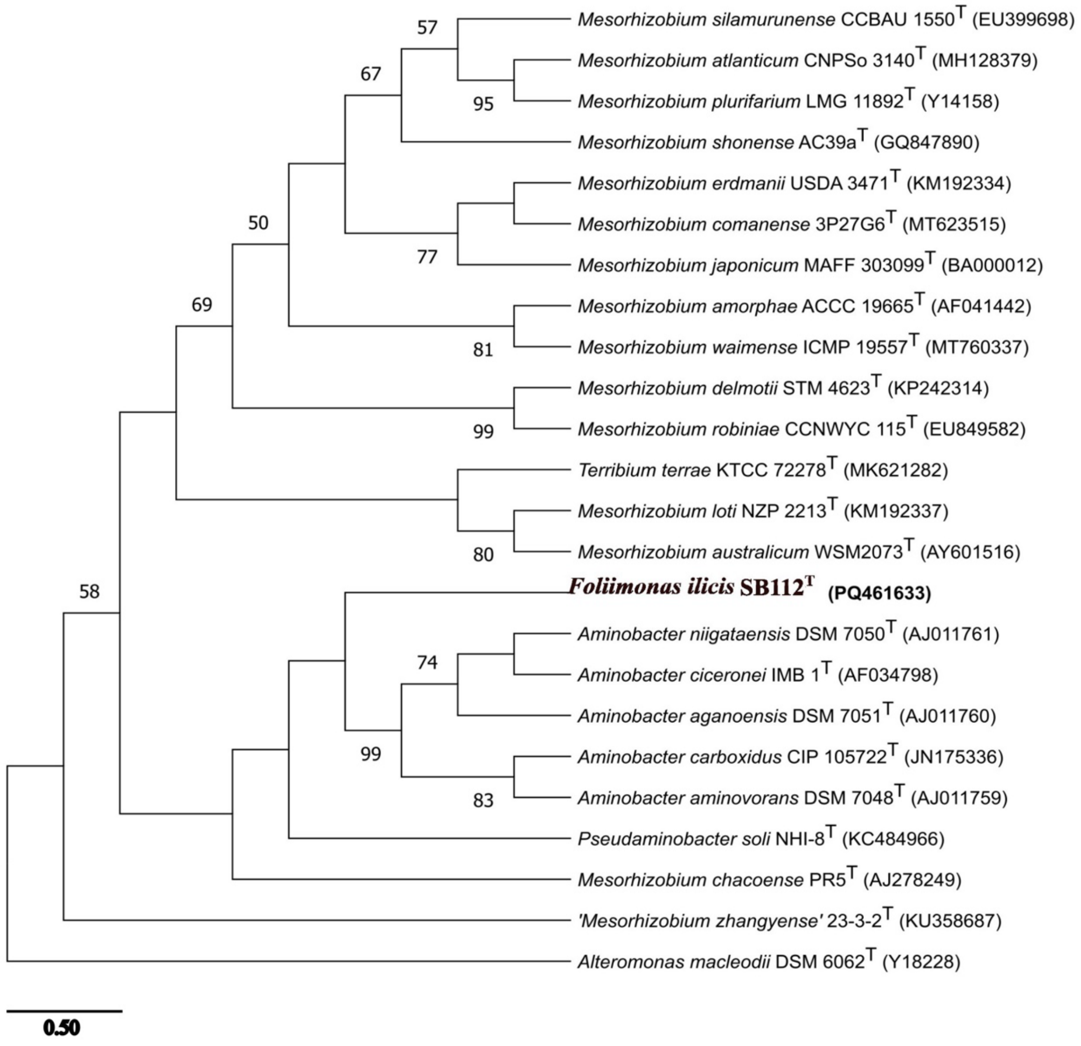

Fig S3:
Heatmap of cpAAI values between *Foliimonas ilicis* SB112^{T} and representative members of the family *Rhizobiaceae*.

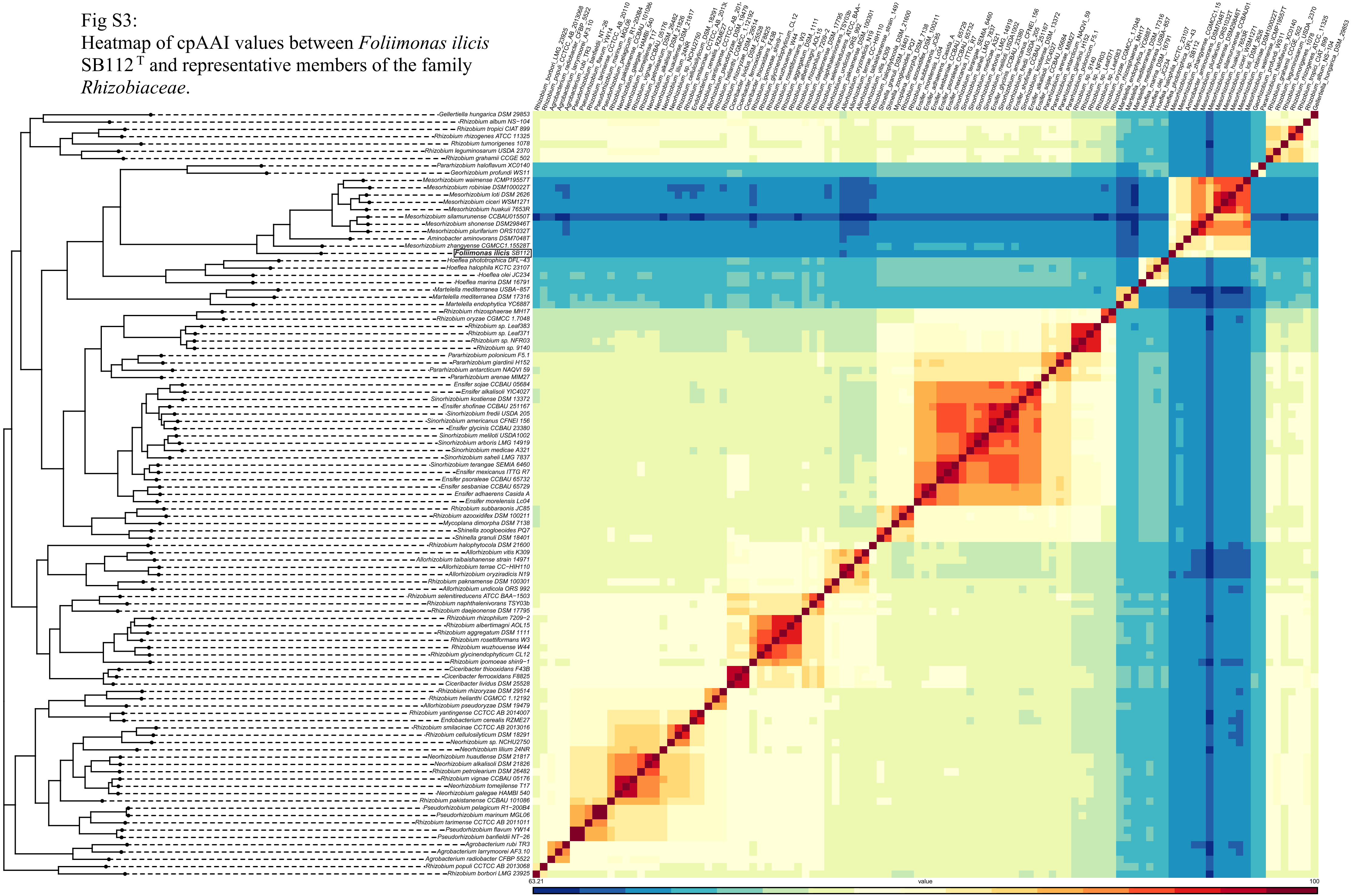

Fig S4:
Subsystem coverage and category distribution of whole genome. The pie chart indicates the counts of each subsystem feature and the subsystem coverage. The number of genes in each subsystem category was shown in brackets.

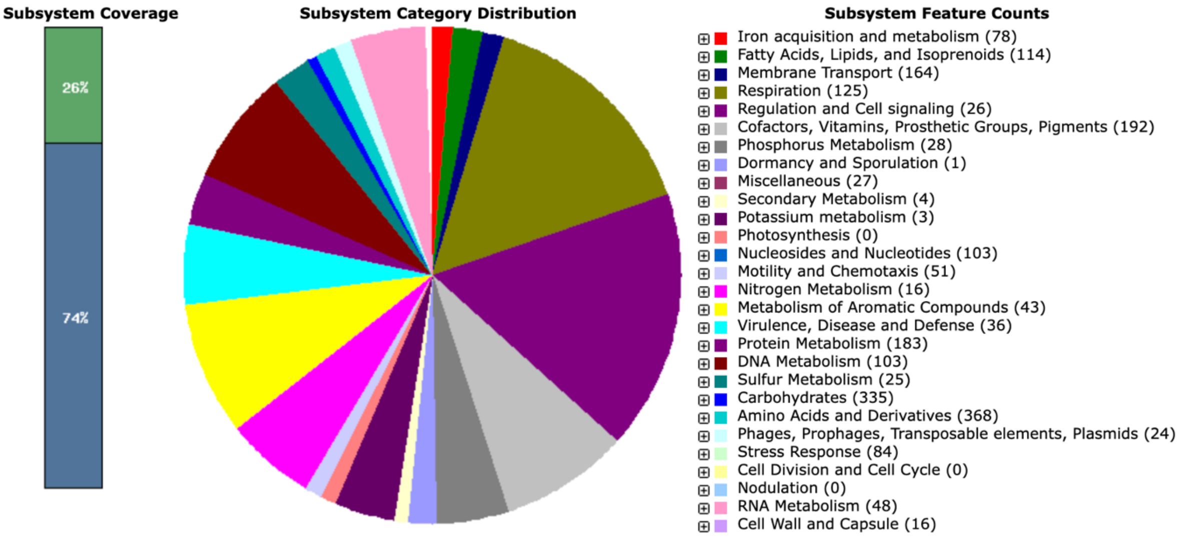

Fig S5:
Two-dimensional TLC of the total polar lipids of strain SB112^{T}

DPG = Diphosphatidylglycerol

PG = Phosphatidylglycerol

PC = Phosphatidylcholine

AL = Aminolipid

PL = Phospholipid

L = Lipid

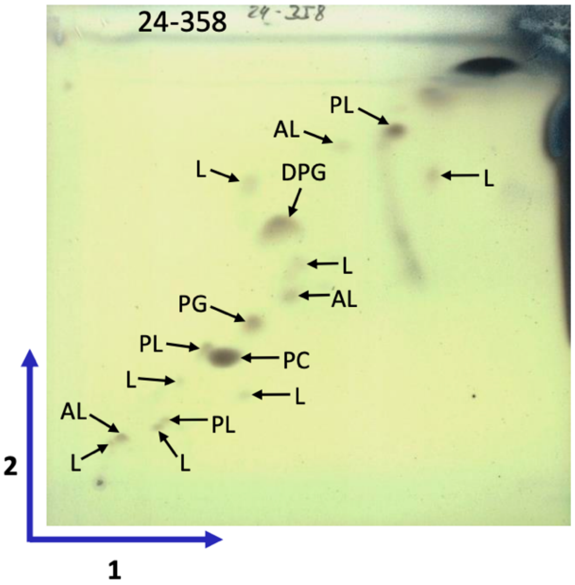

Table: S1
16S rRNA gene sequence similarity, digital DNA-DNA hybridization (dDDH), genome to genome distance calculator (GGDC), average nucleotide identity (ANI), and average nucleotide identity blast (ANIb) values between SB112 ^{T} and phylogenetically closest members

**Species**	**Strain**	**Accession number**	**ANIb (%)**	**OrthoANI (%)**	**GGDC distance**	**dDDH (%)**
Mesorhizobium zhangyense	CGMCC 1.15528^{T}	JAAKZG000000000	74.91	76.3285	0.2074	21.2
Mesorhizobium silamurunense	CCBAU 01550^{T}	JHQR00000000	73.52	74.6478	0.2203	19.9
Mesorhizobium waimense	ICMP 19557^{T}	QZWZ01000000	73.44	74.7023	0.2173	20.2
Mesorhizobium plurifarium	ORS 1032^{T}	CCND00000000)	73.43	74.8275	0.2151	20.4
Mesorhizobium robiniae	DSM 100022^{T}	JBEPMC000000000	73.42	74.7698	0.2151	20.4
Mesorhizobium shonense	DSM 29846^{T}	JBEPLM000000000	73.36	74.7679	0.2151	20.4
Aminobacter aminovorans	DSM 7048^{T}	SLZO00000000	73.22	74.5580	0.2187	20.1
Mesorhizobium loti	DSM 2626^{T}	QGGH00000000	69.87	74.7849	0.2194	20.0

Table S2:
Pairwise cpAAI values between *Foliimonas ilicis* SB112 ^{T} and representative *Rhizobiaceae* strains (dataset of Kuzmanović et al., 2022, supplemented with closest relatives of SB112 ^{T})

Attached as an Excel file (Table S2).

Table S3: Average amino acid identity (AAI) values calculated using FastAAI to assess the genomic relatedness of *Foliimonas ilicis* SB112 ^{T} to its closest relatives.

**Query**	**Target**	**Average Jaccard similarity**	**Number of shared single-copy proteins (SCPs)**	**Average Amino Acid identity**
Foliimonas ilicis SB112 ^{T}	*Aminobacter aminovorans* DSM 7048^{T}	0.4498	78	65.25
Foliimonas ilicis SB112 ^{T}	*Foliimonas ilicis* SB112 ^{T}	1	78	>90%
Foliimonas ilicis SB112 ^{T}	*Mesorhizobium loti* DSM 7048^{T}	0.4401	78	64.77
Foliimonas ilicis SB112 ^{T}	*Mesorhizobium plurifarium* ORS1032^{T}	0.4313	78	64.33
Foliimonas ilicis SB112 ^{T}	*Mesorhizobium robiniae* DSM 100022^{T}	0.4432	78	64.92
Foliimonas ilicis SB112 ^{T}	*Mesorhizobium shonense* DSM 29846^{T}	0.4313	78	64.33
Foliimonas ilicis SB112 ^{T}	*Mesorhizobium silamurunense* CCBAU 01550^{T}	0.4334	78	64.44
Foliimonas ilicis SB112 ^{T}	*Mesorhizobium waimense* ICMP 19557^{T}	0.4383	78	64.68
Foliimonas ilicis SB112 ^{T}	*Mesorhizobium zhangyense* CGMCC 1.15528^{T}	0.4878	78	67.13
